# Culturally adapted post-diagnostic dementia support for South Asian people living with dementia and caregivers: a rapid review

**DOI:** 10.1093/ageing/afag266

**Published:** 2026-09-10

**Authors:** Amirah Akhtar, Emmanuel Nwofe, Emily Dodd, Gary Fry, Nerice Knight, Karan Jutla, Richard Cheston, Sahdia Parveen

**Affiliations:** Department of Applied Dementia Studies, University of Bradford, Bradford, England, UK; Department of Applied Dementia Studies, University of Bradford, Bradford, England, UK; School of Health and Social Wellbeing, University of the West of England, Bristol, England, UK; Department of Applied Dementia Studies, University of Bradford, Bradford, England, UK; Herefordshire and Worcestershire Health and Care NHS Trust, Worcester, England, UK; Faculty of Education, Health, and Wellbeing, University of Wolverhampton, Wolverhampton, England, UK; School of Social Sciences, University of the West of England, Bristol, England, UK; Department of Applied Dementia Studies, University of Bradford, Bradford, England, UK

**Keywords:** Alzheimer, culture, dementia, post-diagnostic support, South Asian, older people

## Abstract

**Background:**

The number of minority ethnic people living with dementia (PLWD) in the UK is predicted to rise to 50 000 by 2026 and 172 000 by 2051. As the global population ages, there is a greater need to develop culturally appropriate post-diagnostic support for PLWD from minority ethnic backgrounds.

**Methods:**

A rapid review was conducted of culturally adapted post-diagnostic dementia support for South Asian people with dementia and carers. Eight electronic databases were searched from inception until 16 September 2025. Databases included Cumulative Index to Nursing and Allied Health Literature, Excerpta Medica Database, MEDical Literature Analysis and Retrieval System Online, Psychological Information, Turning Research Into Practice, Allied and Complementary Medicine Database, Social Policy and Practice and the Cochrane Database of Systematic Reviews. Two reviewers independently screened the studies. Consistent with rapid review methods, no formal quality assessment of included studies was undertaken. The rapid review adhered to Preferred Reporting Items for Systematic Review and Meta-Analysis guidelines.

**Results:**

Twelve studies were included. These included seven carer support programmes focusing on raising awareness and education on dementia and care. These interventions increased carers’ knowledge of dementia and confidence in caregiving. Four studies reported on psychosocial interventions: Cognitive Stimulation Therapy, Cognitive Behaviour Therapy and Meditation Therapy, demonstrating benefits for caregiver burden and mental health. One study reported on service-level innovations through a South Asian link nurse, which improved access to services and facilitated the development of culturally appropriate information materials.

**Conclusion:**

The findings of this rapid review demonstrate the feasibility and perceived value of culturally sensitive psychoeducation, carer training and psychosocial interventions. However, research remains small-scale, methodologically limited, with little focus given to interventions directly supporting PLWD.

## Key Points

- Lack of research on post-diagnostic support targeted at people living with dementia from South Asian backgrounds.
- Culturally adapted post-diagnostic support demonstrates improvement in carer burden and mental health.
- While culturally tailored educational programmes appear feasible and acceptable, few have been scaled or integrated into routine practice.

## Background

Dementia is a major global public health challenge, currently affecting an estimated 55 million people worldwide and projected to nearly triple by 2050 as populations age [1]. Forecasts suggest a seven-fold rise in dementia prevalence among UK South Asian populations by 2051, far outpacing the projected increase among the White British population [2]. With the rising prevalence, there is an urgent need to ensure equitable access to timely diagnosis and appropriate post-diagnostic support for all populations. In the UK, inequalities in dementia care have long been documented, particularly for people of South Asian backgrounds [3, 4].

Despite this growing need, research repeatedly shows that South Asian people living with dementia (PLWD) and their carers face structural, cultural, linguistic and service-level barriers that limit access to care [5, 6]. Help-seeking is often delayed due to stigma, limited awareness of symptoms and misunderstandings about dementia as a normal part of ageing [7, 8]. Much of the existing literature has focused on pre-diagnostic issues such as knowledge, attitudes and perceptions of dementia within South Asian communities [6, 9]. While this work has been important in highlighting why diagnosis may be delayed, it has resulted in comparatively limited attention being given to what happens after a diagnosis is made.

Post-diagnostic support is crucial to enable people with dementia and their families to live well. However, much of the current evidence base for the effectiveness of post-diagnostic support interventions often fails to capture experiences of people from minority ethnic backgrounds. This means that little is known about what materials, interventions or best-practice models have been developed or adapted for South Asian communities either in the UK or internationally. The small number of existing reviews relevant to this area have tended to focus on dementia awareness, symptom recognition or experiences of stigma within South Asian groups [9, 10]. These have generated important insights but have not systematically mapped the landscape of post-diagnostic resources, culturally adapted materials or evidence-based interventions designed to support PLWD and carers beyond the point of diagnosis. Given the projected increase in dementia prevalence among South Asian communities and the ongoing commitment to reduce inequalities as outlined in national dementia strategies, this represents an important and urgent gap.

Rapid reviews are particularly suited to emerging priority areas where evidence is diffuse, heterogeneous or not yet well consolidated, enabling timely insights to inform practice. This rapid review aims to identify existing materials, resources and interventions that have been developed or culturally adapted to support PLWD from South Asian communities, examine the evidence base underpinning them and highlight gaps in provision. For the purpose of this review, we define cultural adaptation as ‘the systematic modification of an evidence-based treatment or intervention protocol to consider language, culture, and context in such a way that it is compatible with the client’s cultural patterns, meanings, and values’ [11]. By mapping what is currently available and identifying areas of unmet need, this review seeks to contribute to the development of equitable, culturally responsive post-diagnostic support for South Asian PLWD and their carers.

### Review questions

1) What materials, resources and best practices have been developed or culturally adapted to enable people with dementia and their carers from South Asian communities to live well and be supported with a diagnosis of dementia?
2) What is the evidence base for these materials and processes?
3) What and where are the gaps in the provision of culturally adapted materials, resources and best practices for PLWD and their carers from South Asian Communities?

## Method

A rapid review methodology was used to identify literature to answer the review questions. No formal quality appraisal was undertaken. This decision was guided by the rapid review methodology, the exploratory aim of mapping culturally adapted post-diagnostic support and the inclusion of heterogeneous development and cultural adaptation studies, which are not easily comparable using standard critical appraisal tools [12].

This rapid review followed the Preferred Reporting Items for Systematic Review and Meta-Analysis (PRISMA) approach (see Supplementary Appendix 1 in the Supplementary Data for the full details of the PRISMA). The search of the literature was conducted via electronic databases including Allied and Complimentary Medicine Database, Cumulative Index to Nursing and Allied Health Literature, Excerpta Medica Database, MEDical Literature Analysis and Retrieval System Online, Psychological Information, Turning Research Into Practice, Social Policy and Practice and the Cochrane Database of Systematic Reviews. The search strategy was developed by the team and a subject librarian. Subject headings and free-text words were identified for dementia, South Asian communities and post-diagnostic support. All databases were searched from inception until 16 September 2025. We searched grey literature, including thesis databases and reference lists of relevant reviews and included studies; however, due to the nature of rapid reviews we were unable to conduct an extensive search of grey literature. The search strategy is presented in Supplementary Appendix 2 of the Supplementary Data.

### Eligibility

Eligible studies in this rapid review reported on PLWD and/or carers of PLWD from South Asian communities and/or health and social care professionals working with PLWD from South Asian communities. The countries considered to be part of South Asia included Afghanistan, Bangladesh, Bhutan, India, the Maldives, Nepal, Pakistan and Sri Lanka (SAARC). Search terms were intentionally left wide as studies conducted on South Asian populations internationally, in majority and minority South Asian contexts were deemed to produce useful insights. Eligible studies discussed, outlined, proposed or evaluated processes, tools, materials, service models or guidelines developed to assist or improve the care and support for PLWD in community settings. Studies reporting on non-dementia topics (e.g. mild cognitive impairment, Korsakoff syndrome, delirium) or if they were conducted in residential or institutional settings (e.g. nursing homes and acute hospitals) were excluded. Only studies published in English were included due to time constraints.

### Evidence screening and selection

Records were managed and screened using Covidence software version 2.0. Detailed instructions for screening were developed, shared, and agreed upon with the research team to ensure consistency. Four reviewers (E.D., G.F., N.K., A.A., E.N.) conducted double screening of titles, abstracts and full-text. Conflicts were resolved by a third reviewer (S.P.).

### Data extraction

Data were extracted through a structured data extraction sheet created in Covidence by four reviewers (E.D., G.F., A.A., E.N.). Any discrepancies during data extraction were resolved by a third reviewer (S.P.) (see Supplementary Appendix 3 for the full details of the data extraction form).

## Results

**Figure 1:**
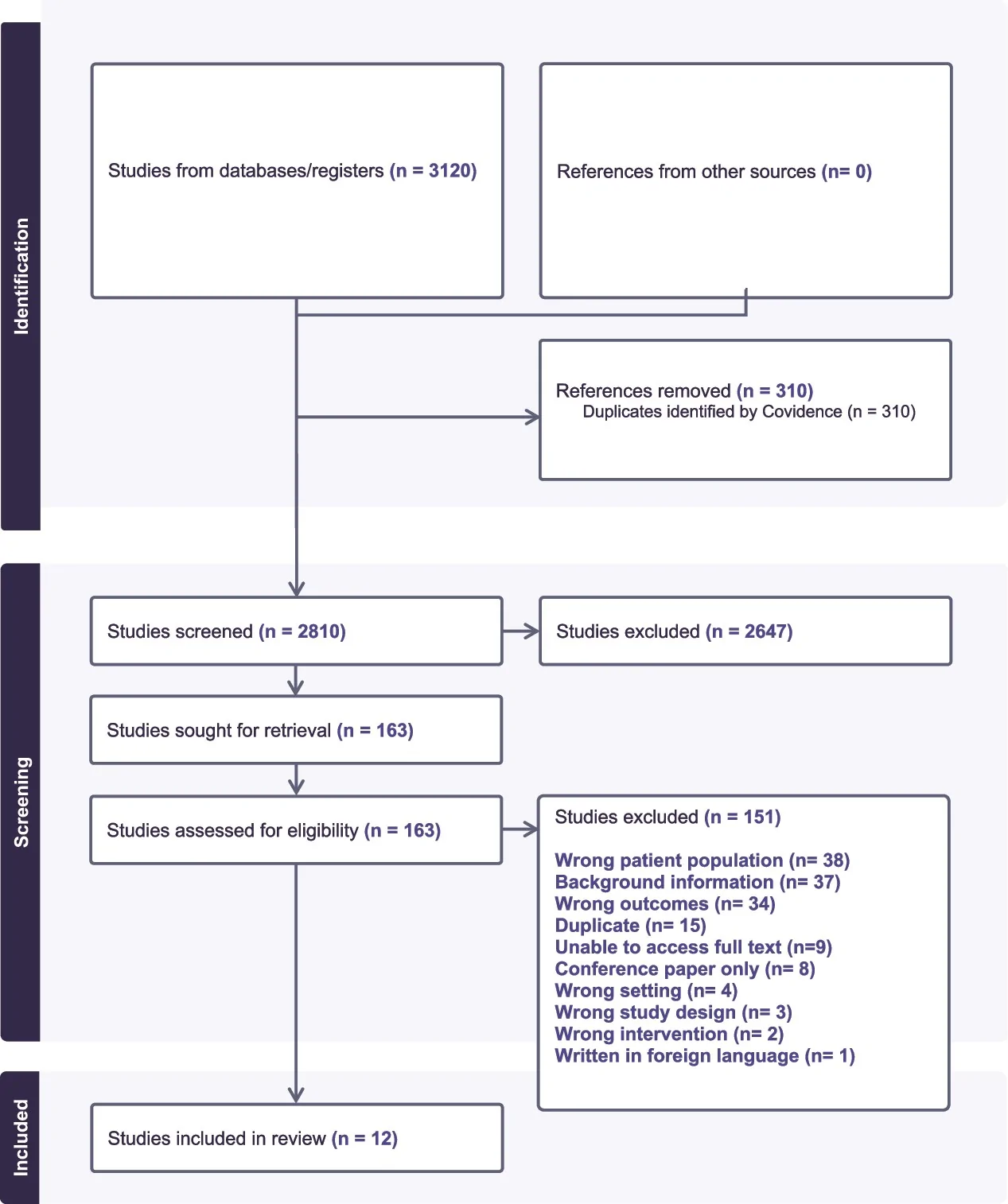
PRISMA flow diagram of 3120 records identified, 310 duplicates were removed, leaving 2810 records screened. A total of 163 records were assessed for full-text eligibility, with 151 excluded. Twelve studies were included in the review.

The search results are detailed in the PRISMA diagram (Figure 1). The database searches retrieved 3120 unique references. Of these, 12 studies were considered eligible for inclusion; see Table 1 for study characteristics. Studies were conducted in the UK (*n* = 4), India (*n* = 5), Pakistan (*n* = 1) and Denmark (*n* = 2). Culturally adapted post-diagnostic dementia support was dominated by interventions designed for carers [13–21], followed by PLWD [22, 23] and one intervention designed to support both PLWD and family carers [24]. Sample sizes ranged from 4 to 110 participants, with women typically comprising the majority of carers.

**Table 1:** Study characteristics.

Author, year	Country	Study design	Details of post-diagnostic support	Cultural adaptations	Sample characteristics	Main findings
Mackenzie (2006)	UK	Qualitative	A tailored 10-week support group programme combined with advocacy support for carers The findings report on semi-structured interviews which explored carers’ experiences of caregiving; the nature and availability of family, community and mainstream service support; knowledge of dementia and what the carers would want from a support group programme	**Terminology:** Due to absence of an equivalent and appropriate term for dementia, the team adopted an alternative approach by advertising the project as ‘Family carers of older people with memory problems’ **Recruitment:** Project advertised in local community and religious settings, local radio broadcasts to advertise the project. A voicemail service was set up in a range of languages for people to contact the project team by telephone **Language:** The intervention was delivered in a range of preferred community languages	11 Pakistani carers and 5 Indian carers. Demographic information not reported	South Asian carers experience a religious obligation to provide care. Dementia has a stigma in South Asian communities, often with magical explanations which could tarnish families across generations. This results in concealment of the condition by those with lived experience, but as the condition develops, carers are forced into isolation in community, using few services
Dias *et al*. (2008)	Goa, India	RCT	Home care programme for supporting caregivers of person with dementia in developing countries **Content:** Aimed to improve the awareness and knowledge of family caregivers regarding dementia, to provide emotional support to caregivers, to maximise their caregiving resources and to improve their caregiving skills **Duration:** The minimum frequency of visits was at least once a fortnight for 6 months **Delivery team:** Two full-time Home Care Advisors. One local psychiatrist. One lay counsellor **Control group:** Dyads received only education and information regarding dementia and were then placed in a waiting list to receive the intervention after 6 months	**Translation:** Study instruments were translated into Konkani, the local language of Goa, using standard methods of translation and back-translation **Setting:** Intervention was delivered in community and home-based settings, since many people with dementia and their families had difficulties accessing public health services **Language:** Delivered by Home Care Advisors who knew the local language	Total sample: 81 PLWD and their carers **Intervention group** Total sample: 41 **Mean age** PLWD: 79.4 Carers: 53.2 **Gender** PLWD: 26 male Carers: 4 male carers **Education** PLWD: 16 below primary education PLWD Carers: 8 below primary education. **Control group** Total sample: 40 **Mean age** PLWD: 77.3 Carers: 53.8 **Gender** PLWD: 27 male Carers: 6 male **Education** PLWD: 19 below primary education Carers: 9 below primary education carers	**Primary outcome:** Caregiver mental health (GHQ score) **Secondary outcomes:** Perceived burden (Zarit Burden score) (NPI-D) and severity of the behavioural problems in the person with dementia (NPI-S), and functional ability of the subject [Everyday Abilities Scale for India (EASI)]. Death records were collected for all people with dementia who died during the course of the project and a caregiver GHQ was carried out one month after the death **Completion rate:** 59 completed the trial and 18 died during the trial The intervention led to a significant reduction of mental health issues and behavioural disturbance scores and non-significant reductions in burden, activities of daily living and behavioural problem scores. There was also a non-significant reduction in the total number of deaths in people with dementia in the intervention group
Kaur *et al*. (2010)	UK	Service evaluation	A South Asian link nurse role	A South Asian link nurse role was created specifically for Punjabi speaking people. This was a qualified community psychiatric nurse, who speaks Punjabi, related languages and English, and understands the relevant cultural issues Language: Punjabi and similar local language of the South Asian population	The link nurse had a caseload of 88 clients of Indian background with mixed diagnoses. The ongoing caseload includes new referrals, which have varied from 25 to 40 per year, and others from previous years	The nurse promoted knowledge, understanding and sensitivity of cultures; contributed to the development and distribution of information in different formats and languages; provided outreach within communities; made use of vehicles for publicity’ prepared for client and carer involvement; involved communities in the planning process; supported improvements in primary care; encouraged liaison in acute hospital settings; piloted alternative services; improved access to services; supported community and voluntary groups; and helped to ensure day, respite and residential care is culturally appropriate
Aguirre and Werheid (2014)	UK	Process of adapting CST to other cultures	Culturally adapted CST for people with mild-to-moderate dementia	A community-based, bottom up approach was taken to culturally adapt CST. The approach described is based on the Formulative Methods for Adapting Psychotherapy, which includes five stages: Generating knowledge and collaborating with stakeholders Integrating generated information with theory and empirical and clinical knowledge Review of the culturally adapted CST intervention by stakeholders and further revision Testing the culturally adapted version Synthesising stakeholder (participant and facilitator) feedback from pilot and finalising the culturally adapted intervention	The guidelines presented are applicable to several culturally diverse ethnic groups including people of South Asian background with mild-to-moderate dementia	This study provides a series of guidelines to support people to adapt CST to other cultures. The first stage is consultation via focus groups of all stakeholders, from family members to health/mental care practitioners, and people with cultural expertise. Stage two involves synthesis of this material into CST manual form. Stage three involves focus groups with CST facilitators, with feedback from observers in order to finalise the manuals. Stage four involves testing the manual in different settings. Stage five involves further focus groups with facilitators, reflecting on using the CST manuals in practice
Ali and Bokharey (2015)	Lahore, Pakistan	Within-subjects design	Individually tailored CBT for caregivers of dementia **Duration:** 5 and 8 weeks, each lasting 45–60 minutes. 80 sessions in total (3600 hours) with all participants **Content:** Rapport building, psychoeducation, cognitive restructuring, relaxation training, anger management, assertiveness training, stress management, burden management, sleep hygiene and time management and relapse prevention	Specific cultural adaptations not mentioned; however, CBT sessions were individually tailored. The agenda structure was informed by a qualitative, interpretive phenomenological study [26] **Language:** Delivered in Urdu	**Total sample:** 8 **Gender:** 3 male; 5 female. **Mean age:** 75.13 **Education:** Reported that majority of participants were educated to degree level	**Primary outcomes:** **‘Burden Assessment Scale’** **‘General Health Questionnaire-28’** **‘State–Trait Anger Expression Inventory-2’** CBT significantly reduced burden level among dementia caregivers. CBT improved physical and mental health of caregivers. CBT significantly reduced anger among the caregivers
Raghuraman *et al*. (2017)	Chennai, India	Process and pilot of culturally adapting CST	Culturally adapted CST for people with mild-to-moderate dementia	The adaptation process followed the guidelines for international adaptation proposed by Aguirre and Werheid [22] **Phase 1—Generating knowledge and collaborating with stakeholders** Seven key stakeholders: general physician, a geriatric psychiatrist, two trained nurse practitioners, a psychologist, a senior research coordinator, the manager of a dementia day-care centre and a caregiver were invited for a focus group discussion. All were familiar with the culture and language of the setting **Phase 2—Integrating generated information with** **theoretical, empirical and clinical knowledge** **Changes to the structure of sessions:** • Timing of sessions to be held between 11 and 1 p.m. • Transport facilities were offered to participants at a nominal fee • An induction period of 2 weeks was incorporated to help the participants familiarise themselves with the members of staff and setting and to establish a routine • Sessions start with tea and biscuits. Group lunch for bonding Longer sessions of CST preferred (2–3 hours) **Changes to the content:** • In the session ‘Childhood Memories,’ re-creating childhood festivals, celebrations and other memorable events from childhood were included as activities instead of re-creating a childhood bedroom, because the concept of a separate child bedroom does not exist in most Indian families • A traditional, South Indian counting game called ‘Pallanguzhi’ was added to the ‘Number Games’ session • Culturally relevant and appropriate items were generated for some sessions, such as word associations, faces, scenes and team quiz • Meal planning and cooking activities in the ‘Food’ session were replaced with the activity of budgeting for ordering a meal delivery for a specific number of people because cooking a local cuisine could be very complex, and also men from that generation may not be familiar with cooking **Setting:** Dementia day-care centre **Language:** Delivered in Tamil	**Pilot study 1:** **Total sample:** 5 (2 drop outs). **Gender:** All male **Mean age:** 77.2 **Education:** On average 13.6 years of schooling **Pilot study 2:** **Total sample:** 4 (1 drop out) **Gender:** all male **Mean age:** 75.5 **Education:** On average 11.75 years of schooling	**Pilot study 1:** Feedback from pilot study 1 indicated a need to discard of a group song as it was deemed to be childish. This was replaced by popular local songs being played in the background Unfamiliar activities (gardening, knitting) were replaced by making flower garlands, and rangolis (colourful patterns made using special powders) were used in the ‘Being Creative’ session In the ‘Using Money’ session, participants were not interested in just guessing the prices, they were asked to compare prices of commodities between different time periods These changes were incorporated and tested in pilot study 2. Statistical analyses were not conducted due to small sample size **Participant feedback:** Participants reported looking forward to sessions. In pilot 1 participants reported that 2 hours in the venue felt too rushed and short. In pilot 2 time was increased to 4 hours, no complaints were reported **Caregiver feedback:** Caregiver gave positive feedback. Some reported observable improvements in the language/communication, social interaction and overall quality of life of persons with dementia. Elder-friendly venues with accessible elevators, railings and toilets were suggested **Facilitator feedback** Delivering CST was described as being enjoyable. Some sessions such as ‘Being Creative’ were challenging, due to lack of interest or sessions being perceived as ‘childish’. The role of co-facilitator in engaging and involving all participants in the sessions was vital
Parveen *et al*. (2018)	UK	Qualitative (evaluation)	Information Programme for South Asian Families **Content:** Addressing understanding dementia, legal and money matters, looking after others and looking after yourself **Delivery:** By an Alzheimer’s Society facilitator in partnership with a local South Asian community organisation Duration: 4 sessions	**Language:** Delivered in South Asian languages **Culturally appropriate data collection methods:** **Knowledge quiz:** A social quiz was designed to overcome the linguistic and cultural barriers associated with a written questionnaire. Participants held up coloured numbered cards to show how much they agreed with each of six statements. The cards represented a Likert scale, from 1 (strongly disagree) **Focus groups:** The focus groups were facilitated by two researchers, one of whom was multilingual, and discussion occurred in the language choice of participants (mainly Punjabi, Hindi, Urdu and English)	42 participants of Indian, Pakistani and Bangladeshi background involved in focus groups 17 participants (including 4 Pakistani and 3 Indian) involved in family interviews	The information programme enhanced understandings of dementia and helped participants to become more aware of service support available. This resulted in enhanced confidence in the care they provided. Some carers felt less isolated after meeting other carers during the programme. PLWD also benefitted as carers grew more able
Pandya (2019)	Mumbai, India and Kathmandu, Nepal	5-year follow-up, RCT	Customised meditation programme for caregivers **Duration:** 45-minutes guided lesson conducted once a week by instructors and the same was to be practiced at home by the participants once a week prior to the next class	Two meetings were held in Mumbai to design the meditation programme. This was supported by meditation training experts who have customised programmes for diverse clientele. This resulted in the following content: **-**Postures interspersed with relaxation -Slowness in movements -Continuity; inner watchful awareness -Feeling of changes in breathing, heartbeat, blood flow and the resonance of sound -Recognition of linear, surface, 3D and all-pervasive awareness **Language:** The language which the intervention was delivered in is not reported. Measures were delivered in English	**Pre-test: 185** **Control: 89** **Gender:** 12 male; 77 female **Mean age:** 52.5 **Education:** High school: 26 College and higher education: 63 **Intervention: 96** **Gender:** 18 male; 78 female **Mean age:** 52.68 **Education:** High school: 18 College and higher education: 78 **Post-test: 145** **Control: 67** **Gender:** 8 male; 59 female **Mean age:** 57.6 **Education:** High school: 15 College and higher education: 52 **Intervention: 78** **Gender:** 11 male; 67 female **Mean age:** 58.02 **Education:** High school: 14 College and higher education: 64	**Primary outcomes:** Zarit Burden Interview, Revised Caregiving Self-Efficacy Scale, Resilience Scale for Adults and Caregiver Resilience Scale **Intervention group:** Caregivers reported lower perceived caregiving burden, higher self-efficacy in obtaining respite, responding to disruptive patient behaviours and controlling upsetting thoughts, and greater resilience, post-test, in comparison to the control group Gender and relationship with the patient were two strong moderators determining programme impact. Caregiver women, spouses, Hindus, middle class, with college and higher education, homemakers, who attended at least 75% of the meditation lessons and regularly practiced at home (i.e. once weekly for at least 75% of the weeks) reported lower post-test perceived caregiving burden, higher self-efficacy and resilience. Home practice was the strongest predictor of post-test scores. Overall the meditation programme was found to be an effective intervention
Baruah, Loganathan *et al*. (2021a)	India	Description of the process of adaptation of the generic iSupport programme to the Indian cultural setting	An online training course for carers: iSupport programme adapted to the Indian cultural setting	**iSupport developed by WHO adapted to Indian cultural setting** **Cultural adaptation framework:** Barrera and Castro (2006) framework for cultural adaptations and the WHO standardised guide for translation and adaptation **Adaptations:** Words and expressions changed according to local language. For example, food names changed from ‘pasta’ to ‘daal’ Names were changed according to local Indian names, while capturing the cultural and religious diversity. For example, ‘Rekha’ and ‘Iqbal’ Caregiving was mostly family based, therefore resources were changed from ‘nurses/paid help’ to family caregivers (son, daughter and husband) Caregiving scenarios adapted to Indian context. For example, ‘baking cooking’ changed to baking Indian sweets and snacks Audio for exercises was re-recorded with an Indian voice as opposed to a western voice/accent **Language:** Delivered in English	**Face to face interviews:** **Total sample:** 4 **Average age:** 35.75 **Gender:** 3 male; 1 female **Education:** Mean education in years: 16.50 **Online test-run** **Total sample:** 11 **Average age:** 40.64 **Gender:** 6 males, 7 female **Education:** Mean education in years: 16.54	The results of the feedback showed that development of the online training and support programme was appreciated and acknowledged by the participants as an important intervention for caregivers of people with dementia. Some of the aspects which garnered positive response from the participants include—content and information provided in the programme, relaxation exercises provided at the end of each lesson, interactive format of the programme. The participants found the content to be culturally appropriate and relevant to the Indian context. There were a few areas where modifications were required—e.g. changing resource links from global to Indian sources, rewording of emails more sensitively, inclusion of time estimation for completing the course, enlarging fonts
Baruah, Varghese, *et al*. (2021b)	India	RCT	iSupport adapted to Indian cultural setting. **Content:** (1) What is dementia? (one lesson), (2) being a caregiver (four lessons), (3) caring for me (three lessons), (4) providing everyday care (five lessons) and (5) dealing with behaviour changes (10 lessons) **Control group:** Education only comparison condition	See Baruah *et al*. (2021a)	**Intervention:** **Total sample (completers):** 55 **Mean age:** 74.62 **Gender:** 13 male; 42 female **Education (Bachelors or higher):** 26 **Control:** **Total sample (completers):** 26 **Mean age:** 70.35 **Gender:** 14 male; 12 female **Education (Bachelors or higher):** 26	**Primary outcome:** Perceived subjective burden as measured by the ZBI Depression as measured by the Centre for Epidemiological Studies Depression-10 item scale (CES-D10) No significant differences were found between two groups at 3 month follow-up on primary outcomes depression and burden **Secondary outcome:** Person-centred attitude as measured by the Approaches to Dementia Questionnaire—Person-centred attitude subscale Self-efficacy as measured by the RIS Eldercare Self-efficacy Scale. Mastery as measured by the Mastery Scale. Self-rated health as measured by the EuroQol—Visual Analogue Scale Significant improvement was only seen in caregivers’ person-centred attitude towards persons with dementia in the intervention group **Feasibility:** **44.67% recruitment rate** **36.42% study retention rate** **Programme engagement:** 31% of the caregivers in the intervention group completed the recommended number of five lessons; however, 45% did not finish any of the lessons of iSupport
Nielsen *et al*. (2022b)	Denmark	Pilot feasibility trial	**Intervention:** PCSP **Content:** ‘Carer Diary’, structured follow-up, coaching and referral to available services and education programmes **Duration:** 6 months **Delivered by:** Six ethnic Danish primary care dementia coordinators (all women) aged 38–64 years. Included three registered nurses, two nurse assistants and one occupational therapist who all had specialist training in dementia and professional experience with community-based dementia care **Treatment as usual:** Standard treatment consisted of biannual phone contacts to discuss emerging medical, psychological and social issues. Carer support included referral to activities such as standard carer support group meetings and information sessions provided by the local primary care dementia coordinator or Alzheimer’s Association	**Language:** Interpreting services were available for family carers who did not speak Danish fluently **Cultural competency training:** A team of multicultural link workers with basic theoretical training in dementia were available to primary care dementia coordinators for consultations regarding cultural issues **Person centred approach:** Social, cultural and linguistic carer needs assessed by the PCSP included: activities organised by social, cultural and religious groups or organisations the carer could be interested in; socialising with other carers; suitable social and cultural events; culturally and linguistically appropriate reading materials to develop carer’s dementia care knowledge and skills; access to interpreters when needed; and socialising with friends and family	**Intervention group: 4** **Carer** Mean age: 44.5 Gender: 2 male; 2 female **PLWD** Mean age: 72.5 Gender: 4 female **Treatment as usual: 3** **Carer** Mean age: 39.0 Gender: 1 male; 2 female **PLWD** Mean age: 75.0 Gender: 1 male; 2 female	**Primary outcome:** **‘Caregiver competence: Sense of Competence Questionnaire (SSCQ)’** **‘Health-related quality of life (QoL): 12-item Short Form Health Survey version 2 (SF-12)’** **‘Usage of respite care, aged care services and dementia services was measured using 4- point scales’** Sense of competence in managing dementia and mental well-being increased slightly, as shown by differences in change from baseline scores for the SSCQ and SF-12 MCS, while the intervention had no influence on usage of respite care, aged care services and dementia services **‘Severity of behavioural symptoms and carer distress: Neuropsychiatric Inventory Questionnaire (NPI-Q)’** **‘Satisfaction with primary care support: Quality of Care Through the Patient’s Eyes (QUOTE-elderly) questionnaire-specific part’** A reduction of carer distress from Behavioural and Psychological Symptoms of Dementia and increased satisfaction with primary care support was shown by family carers in the intervention group, when comparing NPI-Q Carer Distress and QUOTE-elderly change from baseline scores with those of family carers in the treatment as usual group **‘Three items were added to inquire about cultural and linguistic appropriateness of services provided’** **‘The 20-item satisfaction questionnaire was rated on a 5-point Likert scale’** All family carers reported great satisfaction with the intervention in post-intervention interviews and highlighted the benefits of the structured and proactive approach, individual coaching and case management **Recruitment rate:** Low participation rates for ethnic minority, only 30% consented to take part **Retention rate:** Between baseline and follow-up was 70% **Intervention fidelity** was high, with all primary care dementia coordinators completing all scheduled home visits and telephone contacts None of the caregivers adhered with the Carer Diary, which they mainly related to practical issues concerning its implementation in everyday care routines and a lack of perceived benefit
Nielsen *et al*. (2022a)	Denmark	Feasibility study	**Culturally tailored dementia information programme** One 2-hour session. During the session, the participants were provided with information about dementia, symptoms, causes, treatment options and options for formal support **Delivered by:** Six facilitators (1 male, 5 females; 2 of Turkish, 3 of Pakistani, 1 of Arabic speaking heritage) and was coordinated and supervised by an experienced community nurse. Facilitators completed basic theoretical training in dementia consisting of ~4 hours of self-study. The study neuropsychologist provided training on dementia in relation to minority ethnic communities	**Multi-cultural link workers as co-researchers** Cultural adaptations developed through a collaborative research process with primary care dementia coordinators and with multicultural link workers representing the main minority ethnic communities in Denmark. Two focus group meetings were arranged with primary care dementia coordinators and multicultural link workers to discuss target group, recruitment strategies, content and delivery of the information sessions, and outcome measures **Setting:** Community **Language:** Delivered in the languages of minority ethnic communities and include culture-specific case descriptions	110 participants took part (Arab, Turkish and Pakistani). Majority middle age and older women with an interest in age-related memory problems, and only a minority were experiencing memory problems or cared for someone with dementia themselves. Only one male participated in the programme sessions	**Knowledge quiz:** There was a significant change in the overall knowledge quiz score and trends for significant changes in the proportion of participants agreeing with the statements ‘Memory loss is the primary symptom of Alzheimer’s’ and ‘Alzheimer’s is a normal part of becoming older, like grey hair and wrinkles’ Post-programme, the number of participants agreeing with the statement: ‘If I had memory problems like Fatma, I would seek help from my doctor’ improved after three of the sessions (60%) and was unchanged after two of the sessions (40%), ‘The majority of people over the age of 80 have Alzheimer’s’ improved after four of the sessions (80%) and worsened after one session (20%), ‘Memory loss is the primary symptom of Alzheimer’s’ improved after four of the sessions (80%) and was unchanged after one session (20%), ‘Alzheimer’s is a normal part of becoming older, like grey hair and wrinkles’ improved after four of the sessions (80%) and was unchanged after one session (20%), ‘Alzheimer’s is a form of insanity’ improved after three of the sessions (60%) and was unchanged after two of the sessions (40%), and ‘If I or someone in my family got Alzheimer’s, I would know where to seek help’ improved after two of the sessions (40%) and was unchanged after three of the sessions (60%) **Qualitative feedback:** Overall, multicultural link workers reported satisfaction with the facilitator training programme, adopted recruitment strategies and content and delivery of the information sessions They perceived that most participants had at least some benefit of participating in the information session and that the dementia information programme could potentially help reduce stigma and increase help-seeking in the longer term Recruitment through established groups or organisations and word- of-mouth was found to be superior to handing out flyers or putting up announcements in community centres. A lack of male participants in the programme was explained by women generally being more interested and active in seeking health-related information Mixing of genders in discussions about health-related issues was found to collide with established cultural norms. Suggested strategies for better reaching male participants included having separate sessions for men or arranging communal dining events incorporating dementia information sessions, as this format was considered more culturally appropriate for mixing of genders Overall, they had found it feasible to conduct sessions with participants of different cultural heritage in Danish. When participants had different language requirements, they had generally been able to accommodate these. Although information sessions at community centres were considered effective for reaching larger audiences, most preferred more intimate settings to facilitate discussions about private issues and addressing misconceptions and stigma

Abbreviations: People Living with Dementia; PLWD.

Three types of evidence were found: seven carer support/information programmes, four psychosocial interventions and one service evaluation.

### Carer support information programme

Culturally adapted interventions for caregivers of a person with dementia included information-based support to improve dementia knowledge and awareness of services. Parveen *et al*. [18] evaluated a culturally adapted Information Programme for South Asian families developed by the Alzheimer’s Society (a major national dementia charity in the UK) in consultation with the South Asian community. The programme included cultural examples that reflected South Asian family experiences and caregiving contexts, delivered in South Asian languages and in partnership with South Asian community organisations. This collaborative approach helped engage families who might otherwise not access mainstream services. Knowledge change was assessed via a social quiz to overcome any linguistic and cultural barriers. The programme was found to increase participants’ knowledge about dementia and of dementia services. Carers valued the element of peer support, which increased their confidence in the carer role. The increase in dementia knowledge led carers to adapt their communication style with the person living with dementia, promoting choice, autonomy and empowerment.

Mackenzie [16] developed a culturally adapted support programme and advocacy. The adaptations were informed by semi-structured interviews with carers about their experiences, beliefs, support needs and delivered in participants’ preferred languages. An equivalent term for dementia was not available in South Asian languages; thus, the term dementia was not used, and instead, the support programme was described as being for ‘family carers of older people with memory problems’. Similar to Parveen *et al*. [18], Mackenzie [16] also worked with trusted leaders and organisations within the South Asian community to promote the programme. The programme was found to be beneficial for carers as a space to discuss previously stigmatised experiences and feelings.

Dias *et al*. [17] reported on a Randomised Controlled Trial (RCT) of a home care programme, modified to fit the Indian sociocultural, health system and economic context of Goa. The intervention aimed to educate family members about dementia and improve caregiving skills. The intervention was delivered by Home Care Advisors, recruited from the local community, who received training in dementia care, spoke the local language and were familiar with local customs and family structures. In addition to this, the study used assessment tools that had been specifically developed or adapted for India, including the Everyday Abilities Scale for India, which measures daily functioning using activities relevant to Indian older adults. The intervention significantly improved the mental health of carers, and a reduction in behavioural problems was reported in PLWD.

A further two studies were conducted in India, reporting on the iSupport intervention originally developed by the World Health Organisation (WHO). Baruah *et al*. [14] reported on the process of culturally adapting iSupport to the Indian cultural setting. The study used the heuristic framework for the cultural adaptation of interventions developed by Barrera and Castro [25]. The standardised guide for translation and adaptation developed by WHO was followed to preserve the core therapeutic components of iSupport while improving its cultural relevance, acceptability and usability for Indian caregivers. Adaptations were informed by a literature review and focus group discussions with family caregivers and dementia care professionals. These consultations highlighted the need to better reflect the realities of family-based dementia care in India, incorporate culturally relevant caregiving scenarios, provide information about locally available resources and consider technical limitations such as internet connectivity.

Language and terminology were replaced with culturally familiar examples. Caregiving examples were revised to reflect everyday Indian family life, while references to formal care services were replaced with examples of support provided by family members and neighbours, reflecting the predominantly family-based model of dementia care in India. Following this study, Baruah *et al*. [15] conducted an RCT of the culturally adapted iSupport. A significant improvement was found in the intervention group for caregiver attitude towards the person living with dementia. However, no significant differences were reported in caregiver burden or depression. This could partly be explained by the low recruitment, retention and engagement rates for the intervention.

The WHO iSupport adaptation largely focused on modifying the intervention content (e.g. names, foods, caregiving examples). The cultural adaptation followed a collaborative development process involving primary care dementia coordinators and multicultural link workers. Adaptations included delivering the programme in participants’ native languages and using a semi-structured discussion format to encourage peer discussion. To improve accessibility, the evaluation quiz was simplified, administered verbally and used the term ‘Alzheimer’s’ instead of ‘dementia’, as the latter was unfamiliar to participants. A significant improvement in dementia knowledge was found post-intervention and appeared to reduce stigma and misconceptions, with facilitators noting the value of community-led, gender-sensitive delivery.

Another study in Denmark by Nielsen *et al*. [20] reported on a pilot feasibility study of a Personalised Carer Support Plan (PCSP). The intervention was culturally adapted via the assessment of carers’ social, cultural and linguistic needs alongside their dementia care needs. The PCSP explored carers’ preferences for culturally relevant activities, involvement with social, cultural and religious organisations, opportunities for social interaction, access to culturally and linguistically appropriate information materials, interpreter support and maintaining connections with family and friends. Similar to Nielsen *et al*. [21] who conducted a feasibility study of a culturally adapted information programme for carers, the intervention also included access to multicultural link workers who provided advice on cultural considerations, while dementia coordinators received training on culturally sensitive practice and strategies for addressing potential cultural issues. Overall, caregivers were satisfied with the intervention, reporting an increased sense of competence in managing dementia, improved mental wellbeing and increased satisfaction with primary care support. Despite some promising results, the recruitment rate for minority ethnic participants was low, and the intervention did not influence usage of dementia services.

### Psychosocial interventions

One study described the process and guidelines of culturally adapting Cognitive Stimulation Therapy (CST) for diverse cultures [22]. A community, bottom-up approach was detailed, based on the Formulative Methods for Adapting Psychotherapy. Details of the cultural adaptation stages are presented in Table 1. Adaptations focused on modifying CST activities, materials and delivery methods to improve cultural relevance while maintaining the core therapeutic structure. For example, activities were modified to include culturally meaningful games and discussions about migration. The programme was delivered by bilingual facilitators and prioritised visual and auditory materials over written tasks where literacy was a concern.

Following the guidelines proposed by Aguirre and Werheid [22], Raghuraman *et al*. [23] culturally adapted CST for PLWD in India. Structural changes included providing transport support and an induction period. Activities were modified to include discussions of childhood festivals and celebrations, incorporating the traditional South Indian counting game ‘Pallanguzhi’. The adapted manual was translated into Tamil and pilot tested. The intervention was well-received by caregivers of PLWD, who could see visible improvements in communication, social interaction and overall quality of life. Facilitators reported positive experiences, despite some challenges of maintaining the interest of the person living with dementia and finding alterative engaging materials.

Ali and Bokharey [13] reported on culturally adapted personalised Cognitive Behaviour Therapy (CBT) for dementia carers in Pakistan. While specific cultural adaptations were not reported, the agenda for personalising CBT in this context was informed by themes identified from an interpretative phenomenological study exploring maladaptive cognitions and physical health of carers of PLWD [26]. The adapted CBT incorporated culturally appropriate psychoeducation about dementia and cognitive restructuring techniques. The intervention reported a significant reduction in caregiver burden and anger, with improvement in physical and mental health.

Another intervention for carers was based on a customised meditation programme by Pandya [19] in India and Nepal. Specific details of how the programme was customised are not reported in detail; however, the programme was designed alongside meditation training experts who had experience working with diverse populations. The adapted programme drew on traditional meditation and mindfulness practices and was further tailored to participants’ characteristics, with findings suggesting that cultural factors such as religion, education, occupation and caregiver role influenced engagement and outcomes. Lower rates of caregiving burden, higher rates of self-efficacy and resilience were reported as a result of the programme.

### Culturally adapted service model

A service evaluation study by Kaur *et al*. [24] reported on the introduction of a South Asian link nurse. The role of the link nurse was to provide language-concordant, culturally sensitive care, acting as a bridge between specialist dementia services and local South Asian communities. Dementia information resources were translated into several South Asian languages, including audio formats. Service environments were also adapted to better reflect patients’ cultural and religious backgrounds through culturally familiar foods, celebrations and activities.

Following implementation of the link nurse role, referrals of South Asian people with dementia increased and the number of patient contacts rose over the evaluation period. The authors also reported improved engagement with dementia services through increased community awareness, the establishment of a culturally specific carers’ support group and enhanced collaboration between specialist services, primary care and community organisations.

## Discussion

The findings of this rapid review demonstrate promising developments in culturally tailored education, psychosocial interventions and community-based service innovations. However, the overall evidence base remains fragmented.

### Cultural adaptations: surface level vs deep structure

Cultural adaptation was most commonly achieved through surface structure changes, such as changing Western names to South Asian names. However, few interventions explicitly engaged with culturally embedded explanatory models of dementia. While Mackenzie [16] explicitly incorporated beliefs about caregiving as a religious duty and perceptions of spirit possession, such engagement with meaning systems was not widespread across the evidence base. This pattern reflects a broader tendency in cultural adaptation research to prioritise surface structure modifications over deep structure changes that address underlying cultural values, beliefs and explanatory frameworks [25, 27].

### Community partnership and relational mechanisms of adaptation

A consistent finding across interventions was the central role of community partnership and culturally concordant facilitation. Engagement with community organisations, faith settings and bilingual or bicultural facilitators was identified as essential to recruitment, trust-building and participation. For example, the South Asian link nurse model [24] and community-facilitated education sessions [21] demonstrate how bicultural intermediaries can bridge systemic barriers and enhance service visibility. These roles mirror wider evidence on ‘link worker’ and ‘cultural navigator’ effectiveness in improving health access among minority ethnic elders [28]. Thus, cultural adaptation may be better understood as a socially embedded process, enacted through relationships, community partnerships and service infrastructures, rather than solely through modification of intervention materials. This indicates that cultural adaptation concerns not only what is delivered, but also who delivers it, where it is delivered and through which social networks it is embedded [29, 30].

### Invisibility of people living with dementia

With the exception of CST [22, 23], most of the identified materials were designed for carers rather than for PLWD. While carer wellbeing is crucial, this imbalance risks overlooking the agency, preferences and psychosocial needs of PLWD. Challenges surrounding the inclusion of PLWD in research have been highlighted in a scoping review by Stewart *et al*. [31], including lack of guidance on how to co-produce care planning with PLWD. The adaptation of interventions such as CST offers a potential route to greater inclusion of PLWD, particularly where activities are designed to be linguistically and culturally resonant. Future work should prioritise co-produced, participatory models that actively involve PLWD in design, adaptation and evaluation [32].

A recurrent theme across studies was the pervasive stigma surrounding dementia within South Asian communities. Early qualitative work such as that of Mackenzie [16] directly addressed stigma through group-based discussion of concealed experiences and the use of alternative terminology such as ‘memory problems’ rather than dementia. Similarly, findings reported by Nielsen *et al*. [21] and Parveen *et al*. [18] underscore how beliefs linking dementia to shame and punishment contribute to delayed help-seeking. These findings are consistent with wider cross-cultural literature demonstrating that stigma, misconceptions and lack of culturally relevant information remain major barriers to timely diagnosis and care across conditions [8, 33]. Community-based and faith-sensitive education initiatives offer evidence that culturally resonant information can challenge stigma and improve literacy. Peer discussion and bilingual facilitation reflect good practice principles identified in global dementia awareness campaigns [34].

### Implications for practice

The findings suggest that effective cultural adaptation extends beyond translation and surface-level modification of intervention materials, and engagement is more consistently achieved through relational and contextual mechanisms. In practice, this highlights the importance of investing in community partnerships that can bridge statutory services and South Asian communities. These roles appear central to building trust, reducing stigma and sustaining engagement over time. In line with this, interventions should be embedded within community infrastructures rather than delivered solely through formal healthcare systems. Such an approach is consistent with the National Health Service (NHS) 10-Year Health Plan’s commitment to shifting care into neighbourhoods and communities, recognising that PLWD benefit from coordinated, accessible support that extends beyond clinical services.

### Evidence gaps and future research

Significant gaps remain in the development, accessibility and evaluation of culturally adapted dementia support for South Asian communities in the UK. Cultural adaptation is frequently presented as a feature of interventions, yet the processes by which adaptations were developed, implemented and evaluated are rarely described in sufficient detail. Consequently, it remains unclear whether positive outcomes are attributable to the intervention itself, the cultural adaptations or the interaction between the two. Limited research has been found on post-diagnostic support targeted at the person living with dementia from South Asian backgrounds. Future research should move towards more inclusive, meaningful, person-centred and participatory approaches that actively involve PLWD in decision-making, which includes setting research priorities.

The results of this review suggest that support delivered by trusted community organisations was well accepted by South Asian communities. However, depending on the type of intervention and skills required, partnership between organisations such as the NHS and community may be required. Future research should explore whether culturally adapted interventions are most effectively delivered within primary, secondary or specialist dementia services, community-based organisations or through partnership models involving both healthcare professionals and community providers. Further investigation is also needed to determine who is best placed to deliver these interventions, balancing the cultural knowledge, trust and accessibility offered by community organisations with the clinical expertise required for more complex dementia interventions. Implementation research evaluating the feasibility, acceptability and sustainability of different delivery models would help identify approaches that are both culturally appropriate and practical to integrate into routine dementia care. Evidence of sustained behaviour change, service uptake or health outcomes is largely absent.

### Strengths and limitations

A strength of this rapid review is that a comprehensive number of databases were searched, without country restrictions. Although a grey literature search was undertaken, it was relatively limited in scope. Culturally adapted post-diagnostic dementia support is frequently delivered by community organisations, charities, local NHS services and voluntary sector providers, where programmes may not be formally evaluated or published in academic journals. Consequently, relevant interventions, service evaluations and reports may not have been identified. Studies in languages other than English were excluded. This may have resulted in missed literature given the nature of the topic on cultural adaptations. Consequently, the findings should be interpreted as providing an overview of current approaches to cultural adaptation rather than evidence supporting any single adaptation method.

## Conclusions

Culturally adapted dementia support for South Asian communities is a much needed area for further and robust research. Evidence to date demonstrates the feasibility and perceived value of culturally sensitive psychoeducation, carer training and psychosocial interventions. However, research remains small-scale and methodologically fragmented. Sustained progress will depend on embedding co-designed, culturally competent approaches within mainstream dementia pathways, ensuring that both PLWD and their carers from South Asian communities can access and benefit from equitable, meaningful post-diagnostic support.

## Supplementary Material

Supplementary_materials_afag266
