# Supplementary material for: Culturally adapted post-diagnostic dementia support for South Asian people living with dementia and caregivers: a rapid review
Source: Age Ageing. 2026 Sep 10;55(9):afag266. doi: 10.1093/ageing/afag266 (PMC13559849; doi:10.1093/ageing/afag266)
Supplement: Supplementary file 1 [file supplementary_materials_afag266.zip › Supplementary_materials_afag266_File004.docx]

**Culturally adapted post-diagnostic dementia support for South Asian people living with dementia and caregivers: a rapid review**

**Appendix 3. Data extraction form**

Author (year)

Setting and Sample

Country

Design

Sample characteristics:

Ethnicity

Sample size

Average age of sample

Gender

Details of post-diagnostic support:

Intervention type

Content

Duration

Delivery

Comparator if applicable

Main findings:

Type of post-diagnostic support

Impact on quality of life, wellbeing measures

Cultural adaptations

Impact on knowledge, awareness, service access
