# Supplementary material for: Culturally adapted post-diagnostic dementia support for South Asian people living with dementia and caregivers: a rapid review
Source: Age Ageing. 2026 Sep 10;55(9):afag266. doi: 10.1093/ageing/afag266 (PMC13559849; doi:10.1093/ageing/afag266)
Supplement: Supplementary file 1 [file supplementary_materials_afag266.zip › Supplementary_materials_afag266_File003.docx]

**Culturally adapted post-diagnostic dementia support for South Asian people living with dementia and caregivers: a rapid review**

**Appendix 2. Search strategy**

MEDLINE:

Population 1 – dementia

(dement*.mp OR exp dementia) OR (Alzheimer*.mp OR exp Alzheimer Disease) OR (fronto temporal dement*.mp OR exp frontotemporal dementia) OR (vascular dement* OR exp multiinfarct dementia) OR (“lewy bod*”.mp OR exp Lewy body OR exp diffuse lewy body disease)

**AND**

Population 2 – South Asian communities:

cultural diversity OR cultur* OR ethnic*OR ethnic minority OR Black Asian Minority Ethnic OR Black Minority Ethnic OR BAME OR BME OR Asian OR south Asian OR Indian OR Pakistani OR Bangladeshi OR Gujarati OR Sikh OR Muslim OR Hindi OR Asian British OR Punjabi

**AND**

Outcomes:

Diagnos* OR Assess* OR “Cognitive assessment” OR Process OR Resource OR Policy OR material OR “best practice” OR Adapt* OR Guideline OR Servic* OR Barrier* OR Interpret* OR Translat* OR Care OR Support OR Intervent* OR Tool OR “Primary care” OR “secondary care”.
